# The Multimodal Serotonergic Agent Vilazodone Inhibits L-DOPA-Induced Gene Regulation in Striatal Projection Neurons and Associated Dyskinesia in an Animal Model of Parkinson’s Disease

**DOI:** 10.3390/cells9102265

**Published:** 2020-10-09

**Authors:** Feras Altwal, Connor Moon, Anthony R. West, Heinz Steiner

**Affiliations:** 1 Center for Neurodegenerative Disease & Therapeutics, Rosalind Franklin University of Medicine and Science, North Chicago, IL 60064, USA; feras.altwal@my.rfums.org (F.A.); anthony.west@rosalindfranklin.edu (A.R.W.); 2 School of Graduate and Postdoctoral Studies, Rosalind Franklin University of Medicine and Science, North Chicago, IL 60064, USA; 3 Stanson Toshok Center for Brain Function and Repair, Rosalind Franklin University of Medicine and Science, North Chicago, IL 60064, USA; connor.moon@rosalindfranklin.edu; 4 Discipline of Neuroscience, The Chicago Medical School, Rosalind Franklin University of Medicine and Science, North Chicago, IL 60064, USA; 5 Discipline of Cellular and Molecular Pharmacology, The Chicago Medical School, Rosalind Franklin University of Medicine and Science, North Chicago, IL 60064, USA

**Keywords:** dopamine, serotonin, gene expression, L-DOPA, antidepressant, striatum, Parkinson’s disease

## Abstract

Levodopa (L-DOPA) treatment in Parkinson’s disease is limited by the emergence of L-DOPA-induced dyskinesia. Such dyskinesia is associated with aberrant gene regulation in neurons of the striatum, which is caused by abnormal dopamine release from serotonin terminals. Previous work showed that modulating the striatal serotonin innervation with selective serotonin reuptake inhibitors (SSRIs) or 5-HT1A receptor agonists could attenuate L-DOPA-induced dyskinesia. We investigated the effects of a novel serotonergic agent, vilazodone, which combines SSRI and 5-HT1A partial agonist properties, on L-DOPA-induced behavior and gene regulation in the striatum in an animal model of Parkinson’s disease. After unilateral dopamine depletion by 6-hydroxydopamine (6-OHDA), rats received repeated L-DOPA treatment (5 mg/kg) alone or in combination with vilazodone (10 mg/kg) for 3 weeks. Gene regulation was then mapped throughout the striatum using in situ hybridization histochemistry. Vilazodone suppressed the development of L-DOPA-induced dyskinesia and turning behavior but did not interfere with the prokinetic effects of L-DOPA (forelimb stepping). L-DOPA treatment drastically increased the expression of dynorphin (direct pathway), 5-HT1B, and zif268 mRNA in the striatum ipsilateral to the lesion. These effects were inhibited by vilazodone. In contrast, vilazodone had no effect on enkephalin expression (indirect pathway) or on gene expression in the intact striatum. Thus, vilazodone inhibited L-DOPA-induced gene regulation selectively in the direct pathway of the dopamine-depleted striatum, molecular changes that are considered critical for L-DOPA-induced dyskinesia. These findings position vilazodone, an approved antidepressant, as a potential adjunct medication for the treatment of L-DOPA-induced motor side effects.

## 1. Introduction

Levodopa (L-DOPA) remains the gold standard treatment for Parkinson’s disease (PD) since its introduction in the 1960s [1,2]. As a dopamine precursor, L-DOPA helps restore the disrupted dopamine tone in the basal ganglia in PD patients. However, after 4 to 10 years of L-DOPA treatment, 50–90% of patients experience abnormal hyperkinetic motor complications termed L-DOPA-induced dyskinesias ([3]; reviewed in [4,5]), which severely limit the usefulness of this treatment.

A large literature has related the development of these motor complications to various presynaptic and postsynaptic neuronal changes in the striatum produced by the loss of dopamine terminals and the L-DOPA treatment (e.g., [6,7,8,9,10]). For example, presynaptic changes include large pulsatile fluctuations in striatal dopamine levels following intermittent L-DOPA treatment due to disrupted control of dopamine release and clearance [11,12,13,14]. Studies have revealed that these non-physiological extracellular dopamine levels seen after L-DOPA administration originate from serotonin terminals in the striatum, which, following severe dopamine denervation, become the primary source for dopamine release [14,15,16,17,18,19].

Postsynaptic changes include a variety of structural and molecular adaptations in striatal projection neurons (medium spiny neurons, MSNs), following dopamine denervation and L-DOPA treatment [20,21]. These neuronal changes comprise “supersensitive” postsynaptic (D1) dopamine receptors in the direct pathway (striatonigral) MSNs (dMSNs) [22,23,24], which, in the presence of supraphysiological dopamine levels, produce aberrant second messenger signaling [9,24,25], resulting in facilitated neuronal activity (e.g., [26]) and enhanced transmitter release (e.g., [13,27]), as well as dysregulated gene expression [8,9] in these neurons. Studies have shown that L-DOPA treatment after dopamine lesions produces changes in the regulation of thousands of genes in MSNs (e.g., [9,28,29,30]), including those encoding various neuropeptides, receptors, and transcription factors, and several of these have been directly related to the occurrence or severity of L-DOPA-induced dyskinesia (e.g., [31,32,33,34]; [9], for review).

Recent efforts have focused on modulating the serotonin system in an attempt to regulate abnormal dopamine release in the striatum and prevent the development of dyskinesia ([35] for a recent review). Several groups have investigated the use of selective serotonin reuptake inhibitors (SSRIs) and serotonin (5-HT) receptor agonists in combination with L-DOPA treatment (e.g., [18,36,37,38,39,40,41,42,43]). Of interest are especially 5-HT1A receptor agonists (e.g., [14,18,37,38,39,43,44]), which are thought to stimulate 5-HT1A autoreceptors on serotonin neurons in the dorsal raphe nucleus (DRN) and attenuate neuronal activity and, consequently, serotonin (and abnormal dopamine) release from serotonin terminals. Many of the serotonergic agents tested are indeed found to reduce dyskinesia severity in preclinical studies [35]; however, these agents can also attenuate the prokinetic effects of L-DOPA or have other undesirable side effects (e.g., [42,45,46,47]), limiting their clinical usefulness [48].

One drug of recent interest is vilazodone, an antidepressant approved by the U.S. Food and Drug Administration (FDA). Vilazodone combines SSRI properties with 5-HT1A receptor partial agonist activity [49,50,51]. A recent study found that vilazodone co-administered with L-DOPA was sufficient to significantly attenuate dyskinesia scores, without interfering with the prokinetic benefits of L-DOPA [52]. At least some of these antidyskinetic effects were mediated by the 5-HT1A partial agonist property of vilazodone [52].

In the present study, we used the unilateral 6-hydroxydopamine (6-OHDA) lesion model in the rat to further investigate the behavioral and gene regulation effects of vilazodone co-administered with L-DOPA. For one, we focused on the expression of two neuropeptide markers—dynorphin and enkephalin—which are selectively expressed by dMSNs and indirect pathway (striatopallidal) MSNs (iMSNs), respectively [53], as a means to determine the projection neuron subtypes impacted by this treatment. We also assessed the effects of these drugs on the expression of a transcription factor, zif268, and the 5-HT1B serotonin receptor [33], both of which are regulated by dopamine agonists. Changes in gene regulation were mapped throughout the striatum in order to identify the functional domains affected. Our findings demonstrate that a dose of vilazodone that suppresses all major subtypes of L-DOPA-induced dyskinesia in this PD model inhibited L-DOPA-induced increases in dynorphin, zif268, and 5-HT1B expression, but did not affect enkephalin expression, in the dopamine-depleted striatum, or either gene in the intact striatum.

## 2. Materials and Methods

### 2.1. Animals

Adult male Sprague–Dawley rats (225–249 g at the beginning of the study; Harlan, Indianapolis, IN, USA) were housed 2–3 per cage under standard laboratory conditions (12:12 h light/dark cycle, lights on at 07:00 h; with food and water available ad libitum). Experiments were performed between 13:00 and 17:00 h. All procedures met the NIH guidelines for the care and use of laboratory animals and were approved by the Rosalind Franklin University Animal Care and Use Committee (protocol # 17-05; approved 19 April 2017).

### 2.2. 6-OHDA Lesions

Rats received an injection of desipramine HCl (20 mg/kg, i.p.; in 0.9% saline; Sigma-Aldrich, St Louis, MO, USA) 30 min prior to surgery. After being deeply anesthetized with isoflurane vapors (2–5%), they received an infusion of either 6-OHDA (6-OHDA HBr, Sigma-Aldrich; 8 μg/4 μL, in saline containing 0.1% ascorbic acid) or saline/ascorbic acid (sham lesion) into the right medial forebrain bundle, as previously described [33]. The coordinates used were (in mm): AP, −4.3 (from bregma); ML, 1.6; DV, −8.3 (from dura) [54]. 6-OHDA/vehicle was slowly infused at a rate of 0.4 μL/min, and the cannula was left in place for an additional 10 min before being removed.

### 2.3. Forelimb Stepping Test

The effectiveness of the 6-OHDA lesion was evaluated 4 weeks post-surgery by assessing deficits in forelimb movements using a stepping test [55]. In this test, the rat held by an experimenter is moved sideways, with its forelimb on the side opposite to the movement direction touching the bench surface; the rat will perform adjusting steps during this lateral movement, in our settings, typically 10–14 steps. After dopamine depletion, the number of adjusting steps with the forelimb contralateral to the lesion drops to 3 steps or fewer in animals with a >90% dopamine cell loss in the substantia nigra [56,57]. The present study included only 6-OHDA-infused rats that exhibited 3 or fewer adjusting steps. The lesion was further characterized by measuring tyrosine hydroxylase (TH) immunoreactivity in the striatum.

### 2.4. Drug Treatment

Rats were randomly assigned to different treatments. The 6-OHDA-infused rats with stepping deficits were treated with either L-DOPA (LD) (5 mg/kg, i.p., 2 mL/kg; Alfa Aesar, Tewksbury, MA, USA; plus 12.5 mg/kg benserazide HCl, Sigma-Aldrich) or vehicle, 30 min after receiving an injection of vilazodone HCl (VIL) (10 mg/kg, i.p., 2 mL/kg [52]; Cayman Chemical, Ann Arbor, MI, USA; in 10% Cremophor EL in saline, Sigma-Aldrich) or vehicle (Cremophor) (groups 6-OHDA/VIL/LD, *n* = 8; 6-OHDA/Veh/LD, *n* = 6; 6-OHDA/VIL/Veh, *n* = 8; 6-OHDA/Veh/Veh, *n* = 8). The sham lesion group received repeated injections of vehicle (Sham/Veh/Veh; *n* = 8). Animals received these drug treatments once daily on 5 days (Mon–Fri) for two weeks. In week 3, rats were treated on 3 days. Following the last injection, the rat was placed in an open-field apparatus (43 × 43 cm), and turning behavior was recorded with a video camera for 40 min. Rats were killed 1 h after the final treatment.

### 2.5. Behavioral Analysis

In order to assess the prokinetic effects of L-DOPA treatment, the stepping test was performed on the second day (Tue) of weeks 1 and 2, 60 min after L-DOPA administration. L-DOPA-induced dyskinesias (measured as abnormal involuntary movements, AIMs [58]) were evaluated once daily on three days per week (Wed–Fri) during the first 2 weeks [59]. Rats were individually placed in a clear plastic cylinder, and the behavior was videotaped. AIMs were rated by a rater unaware of the treatment using a time-sampling procedure, i.e., during 1-min periods at 30-min intervals 30–180 min after the L-DOPA injection. Three AIM subtypes were assessed, “axial”, “limb” (forelimb), and “orolingual” ([58], for exact definitions). The frequency of these AIM subtypes was rated using a standard scale (0 = absent; 1 = occasional; 2 = frequent with many interruptions; 3 = continuous but interrupted by external sensory stimuli; 4 = continuous, not interrupted by strong sensory stimuli) [31,58]. In addition, the amplitude of AIMs was scored as follows [58]: axial AIMs (1 = lateral deviation of head and neck at approximately 30° angle; 2 = lateral deviation of head and neck at 30° < angle ≤ 60°; 3 = lateral deviation of the head, neck, and upper trunk at 60° < angle ≤ 90°; 4 = torsion of the head, neck, and trunk at > 90° angle, often causing the rat to lose balance), forelimb AIMs (1 = small involuntary movements of the distal forelimb; 2 = movements of low amplitude, causing translocation of both distal and proximal forelimb; 3 = involuntary movements of the whole limb, including shoulder muscles; 4 = strong limb and shoulder movements, often similar to ballism), and orolingual AIMs (1 = small involuntary movements of the orofacial muscles without tongue protrusions; 2 = involuntary orofacial movements of high amplitude with tongue protrusions). Partial scores, such as 0.5, 1.5, 2.5, and 3.5, were given to increase the sensitivity of the rating. Frequency and amplitude scores were multiplied for each monitoring period (i.e., 30, 60, 90, 120, 150, and 180 min), and the values were added up for a total AIM score for each subtype and an overall total for each testing day.

Turning behavior in the open-field test on the last day (week 3) was assessed during 4 sampling periods, at 5–10, 15–20, 25–30, 35–40 min after L-DOPA injection. Tight circling contraversive to the lesion (i.e., towards the left side) and ipsiversive to the lesion was measured (counted as the number of half-turns).

### 2.6. Tissue Preparation and In Situ Hybridization Histochemistry

The rats were killed with CO_2_, and the brain was rapidly removed, frozen in isopentane, cooled on dry ice, and then stored at −30 °C until cryostat sectioning. Coronal sections (12 µm) were thaw-mounted onto glass slides (Superfrost/Plus, Daigger, Wheeling, IL, USA), dried on a slide warmer, and stored at −30 °C. In situ hybridization histochemistry was performed, as described before [33,60]. Oligonucleotide probes (48-mers; Invitrogen, Rockville, MD, USA) were labeled with [35S]-dATP. The probes had the following sequence: dynorphin, complementary to bases 862–909, GenBank accession number M10088; enkephalin, bases 436–483, M28263; zif268, bases 352–399, M18416; 5-HT1B (Htr1b), bases 62–109, NM 022225. Hybridization and washing procedures were as reported [33,61]. The sections were apposed to X-ray film (BioMax MR-2, Kodak) for 3–11 days.

### 2.7. Analysis of Autoradiograms

Gene expression in the striatum was assessed in sections from 3 rostrocaudal levels (“rostral”, approximately at +1.6 mm relative to bregma [54]; “middle”, +0.4; “caudal”, −0.8), in a total of 23 sectors. These sectors are mostly defined by their predominant cortical inputs and thus reflect different functional domains ([61,62]). Eighteen of these sectors represent the caudate-putamen and 5 the nucleus accumbens.

Hybridization signals on film autoradiograms were measured by densitometry (NIH Image; Wayne Rasband, NIMH, Bethesda, MD, USA), as described [63]. Mean densities were corrected for background by subtracting mean density values measured over white matter (corpus callosum). The illustrations of film autoradiograms depicted in the figures are computer-generated images and are contrast-enhanced where necessary.

### 2.8. Tyrosine Hydroxylase Immunohistochemistry

Striatal sections were processed for TH immunohistochemistry to determine the extent of the dopamine depletion, following previously published procedures [33,60,64]. In short, fresh-frozen, thaw-mounted 12 µm sections were first fixed in 4% paraformaldehyde/saline for 10 min. TH immunolabeling was then revealed with a rabbit peroxidase-antiperoxidase (1:500) reaction, followed by a standard 3,3′-diaminobenzidine protocol with nickel intensification. The signal was measured by densitometry.

### 2.9. Statistics

Treatment effects on gene expression, TH, and stepping were determined by two-factor ANOVA (Prism 8.0, GraphPad, San Diego, CA, USA). Newman–Keuls post hoc tests were used to describe differences between individual groups. Effects on AIMs and turning were assessed with Mann–Whitney tests with alpha adjustments.

## 3. Results

### 3.1. Behavioral Effects of Vilazodone

**Figure 1:**
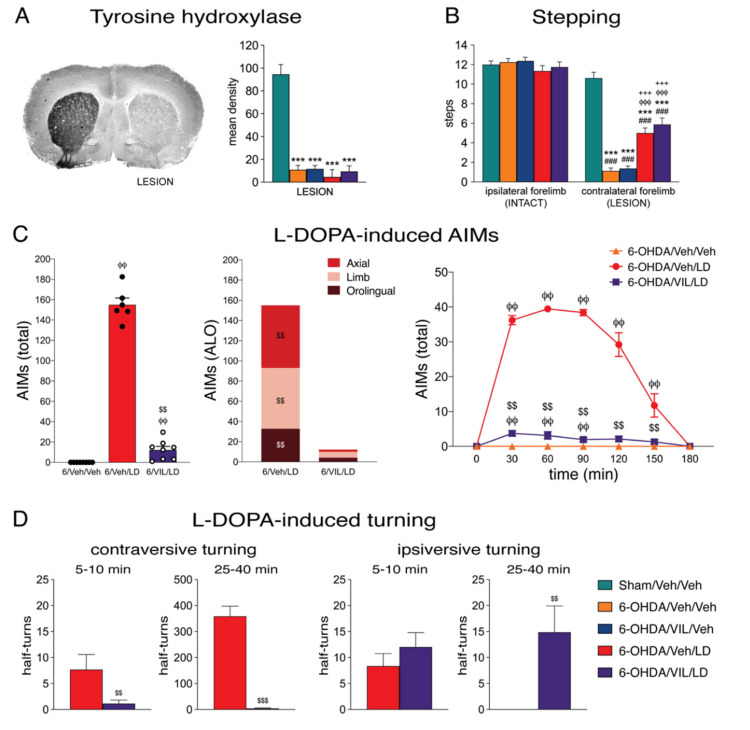
Vilazodone suppressed the development of L-DOPA-induced dyskinesia and turning behavior, but not stepping improvement, after dopamine depletion by 6-OHDA. (**A**) Dopamine depletion (i.e., loss of striatal tyrosine hydroxylase labeling) in the different treatment groups. Representative coronal section from the mid-level striatum labeled with tyrosine hydroxylase immunohistochemistry in a rat with a 6-OHDA lesion on the right side (left) and mean density values (mean ± SEM) for tyrosine hydroxylase labeling on the side of the lesion (% of intact side) (right) are hown for groups that received a sham lesion (Sham/Veh/Veh) or a 6-OHDA lesion followed by repeated vehicle treatments (6-OHDA/Veh/Veh), repeated treatment with vilazodone (10 mg/kg, i.p.) + vehicle (3 weeks) (6-OHDA/VIL/Veh), with vehicle + L-DOPA (5 mg/kg + benserazide, 12.5 mg/kg; 3 weeks) (6-OHDA/Veh/LD), or with vilazodone + L-DOPA (6-OHDA/VIL/LD). (**B**) Stepping test (week 3). The number of steps (mean ± SEM) with the forelimb ipsilateral to the lesion (linked to the intact striatum) and the forelimb contralateral to the lesion (linked to the dopamine-depleted striatum) in the stepping test are given for these treatment groups. L-DOPA improved stepping with the affected (contralateral) forelimb (prokinetic effect); this improvement was not impaired by vilazodone. (**C**) L-DOPA-induced abnormal involuntary movements (AIMs) (week 2; 3-day averages). AIMs (axial, limb, orolingual (ALO); total) scores in the 3-h test are shown for groups with a 6-OHDA lesion followed by vehicle treatments (6-OHDA/Veh/Veh), by vehicle + L-DOPA treatment (6-OHDA/Veh/LD), or by vilazodone + L-DOPA treatment (6-OHDA/VIL/LD). Vilazodone suppressed L-DOPA-induced AIMs. (**D**) L-DOPA-induced turning in the open-field test (week 3). Number of half-turns contraversive (left) or ipsiversive to the lesion (right) during time periods 5–10 min (i.e., before L-DOPA effects appeared in all but one animal) and 25–40 min after L-DOPA injection are given for groups with a 6-OHDA lesion followed by vehicle + L-DOPA treatment (6-OHDA/Veh/LD) or by vilazodone + L-DOPA treatment (6-OHDA/VIL/LD). Vilazodone suppressed L-DOPA-induced contraversive turning but not “spontaneous” ipsiversive turning. ^###^ *p* < 0.001 vs. same group on intact side; *** *p* < 0.001 vs. Sham/Veh/Veh; ^φφ^ *p* < 0.01, ^φφφ^ *p* < 0.001 vs. 6-OHDA/Veh/Veh; ^+++^ *p* < 0.001 vs. 6-OHDA/VIL/Veh; ^$$^ *p* < 0.01, ^$$$^ *p* < 0.001 vs. 6-OHDA/Veh/LD.

Consistent with our previous results [33], 6-OHDA-infused animals that met the inclusion criterion of three or fewer contralateral adjusting steps showed a loss of TH immunoreactivity in the striatum ipsilateral to the 6-OHDA infusion with a range of 88–96% (groups 6-OHDA/Veh/Veh, 6-OHDA/VIL/Veh, 6-OHDA/Veh/LD, and 6-OHDA/VIL/LD; % of intact side; total striatum on middle level; Figure 1A). There was no statistically significant difference between these four groups (*p* > 0.05).

L-DOPA-induced AIMs were assessed on three days per week (Wed–Fri) during the first 2 weeks. Figure 1C shows 3-day averages in week 2 for axial, limb, orolingual, and total AIMs. All L-DOPA only-treated rats (6-OHDA/Veh/LD group) displayed AIMs on the side contralateral to the 6-OHDA lesion; these AIMs lasted for up to 3 h and peaked 60–90 min after L-DOPA injection. Vilazodone co-administration, together with L-DOPA, inhibited the development of AIMs, as AIM counts were significantly reduced in the 6-OHDA/VIL/LD group (Figure 1C). These rats showed less than 10% of total counts compared to those of the 6-OHDA/Veh/LD group. All subtypes of AIMs (axial, limb, orolingual) were reduced by vilazodone (Figure 1C).

Treatment with L-DOPA alone significantly increased the performance in the stepping test (week 3; 6-OHDA/Veh/LD vs. 6-OHDA/Veh/Veh; Figure 1B). In contrast to its impact on AIMs, vilazodone co-administration did not influence these L-DOPA-induced prokinetic effects, as stepping scores in the 6-OHDA/VIL/LD group did not differ from those in the 6-OHDA/Veh/LD group (Figure 1B), nor did vilazodone alone affect stepping (6-OHDA/VIL/Veh vs. 6-OHDA/Veh/Veh).

During the open-field test in week 3, treatment with L-DOPA alone induced high rates of tight contraversive turning (6-OHDA/Veh/LD, half-turns, mean ± SEM, total in the four 5-min sampling periods: 466.3±47.8). This L-DOPA-induced turning behavior emerged between 8 and 20 min after L-DOPA injection and was always accompanied (and often preceded) by the various types of AIMs (not measured). Vilazodone co-administration suppressed contraversive turning (Figure 1D, left). During the 40-min open-field test, rats of the 6-OHDA/VIL/LD group displayed no or very low counts of contraversive turning (total half-turns: 6.1±3.5 vs. 466.3±47.8 in the 6-OHDA/Veh/LD group; *p* < 0.001; data not shown). However, towards the end of their 60-min survival time (in their holding cage and especially during the final CO_2_ exposure), some rats did show tendencies for contraversive turning and, most noticeable, contraversive forelimb AIMs. This turning seemed to have been triggered or enhanced by the handling when rats were transferred back to their holding cage and during the CO_2_ exposure (arousal/stress).

In marked contrast to the contraversive turning, ipsiversive turning was not affected by vilazodone (Figure 1D, right). Seen most frequently at the beginning of the test when rats explored the novel open-field (i.e., before the behavioral effects of L-DOPA set in), ipsiversive turning was present in both 6-OHDA/Veh/LD and 6-OHDA/VIL/LD groups, and these rates did not differ (5–10 min: 6-OHDA/Veh/LD vs. 6-OHDA/VIL/LD, 8.3±2.4 vs. 12.0±2.8; *p* > 0.05; Figure 1D). The 6-OHDA/VIL/LD group displayed low rates of ipsiversive turning throughout the test (25–40 min; Figure 1D), while in the 6-OHDA/Veh/LD group, ipsiversive turning stopped as soon as AIMs and contraversive turning emerged. Overall, animals of the 6-OHDA/VIL/LD group showed an ipsiversive turning bias (ipsiversive/total×100%) of 91.4% (5–10 min) and 79.3% (25–40 min), as compared to the 0% (25–40 min) in the 6-OHDA/Veh/LD group.

### 3.2. Effects of Dopamine Depletion and L-DOPA Treatment on Dynorphin and Enkephalin Expression

**Figure 2:**
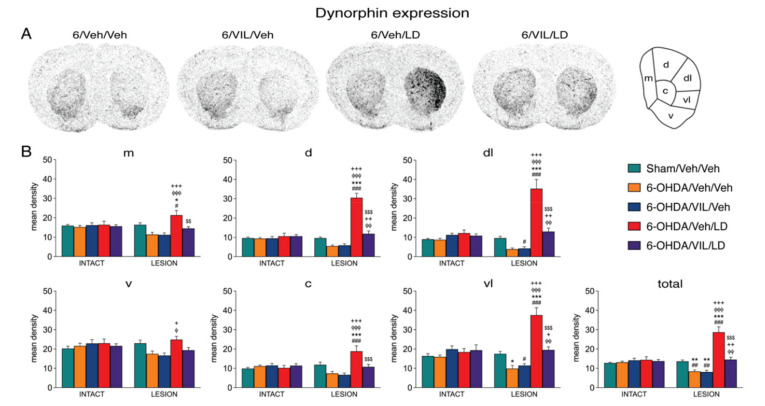
Vilazodone inhibited the L-DOPA-induced increase in dynorphin expression in the dopamine-depleted striatum. (**A**) Illustrations of film autoradiograms depict expression of dynorphin in sections from the mid-level striatum in rats with a 6-OHDA lesion (right hemisphere) followed by repeated vehicle (6-OHDA/Veh/Veh), vilazodone (10 mg/kg) + vehicle (6-OHDA/VIL/Veh), vehicle + L-DOPA (5 mg/kg) (6-OHDA/Veh/LD), or vilazodone + L-DOPA treatment (6-OHDA/VIL/LD). Animals were killed 60 min after the L-DOPA injection. The maximal hybridization signal is in black. (**B**) Mean density values (mean ± SEM) for dynorphin expression in the mid-level striatum on the intact side or the side of the lesion in the sham lesion controls (Sham/Veh/Veh) and the 6-OHDA/Veh/Veh, 6-OHDA/VIL/Veh, 6-OHDA/Veh/LD, and 6-OHDA/VIL/LD groups. Gene expression was measured in six sectors and the total middle striatum (upper right). m, medial; d, dorsal; dl, dorsolateral; vl, ventrolateral; c, central; v, ventral. ^#^ *p* < 0.05, ^##^ *p* < 0.01, ^###^ *p* < 0.001 vs. same group on the intact side; * *p* < 0.05, ** *p* < 0.01, *** *p* < 0.001 vs. Sham/Veh/Veh; ^φ^ *p* < 0.05, ^φφ^ *p* < 0.01, ^φφφ^ *p* < 0.001 vs. 6-OHDA/Veh/Veh; ^+^ *p* < 0.05, ^++^ *p* < 0.01, ^+++^ *p* < 0.001 vs. 6-OHDA/VIL/Veh; ^$$^ *p* < 0.01, ^$$$^ *p* < 0.001 vs. 6-OHDA/Veh/LD.

**Figure 3:**
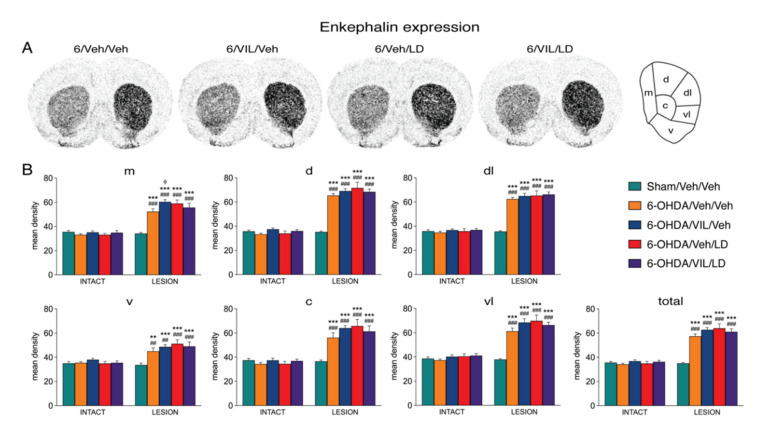
Vilazodone did not alter striatal enkephalin expression after L-DOPA treatment. (**A**) Illustrations of film autoradiograms depict the expression of enkephalin in sections from the mid-level striatum after a 6-OHDA lesion (right hemisphere) followed by repeated vehicle (6-OHDA/Veh/Veh), vilazodone (10 mg/kg) + vehicle (6-OHDA/VIL/Veh), vehicle + L-DOPA (5 mg/kg) (6-OHDA/Veh/LD), or vilazodone + L-DOPA treatment (6-OHDA/VIL/LD). Animals were killed 60 min after the L-DOPA injection. (**B**) Mean density values (mean ± SEM) for enkephalin expression in the mid-level striatum on the intact side or the side of the lesion in the sham lesion controls (Sham/Veh/Veh) and the 6-OHDA/Veh/Veh, 6-OHDA/VIL/Veh, 6-OHDA/Veh/LD, and 6-OHDA/VIL/LD groups. Gene expression was measured in six sectors and the total middle striatum (upper right). m, medial; d, dorsal; dl, dorsolateral; vl, ventrolateral; c, central; v, ventral. ^##^ *p* < 0.01, ^###^ *p* < 0.001 vs. same group on the intact side; ** *p* < 0.01, *** *p* < 0.001 vs. Sham/Veh/Veh; ^φ^ *p* < 0.05 vs. 6-OHDA/Veh/Veh.

**Figure 4:**
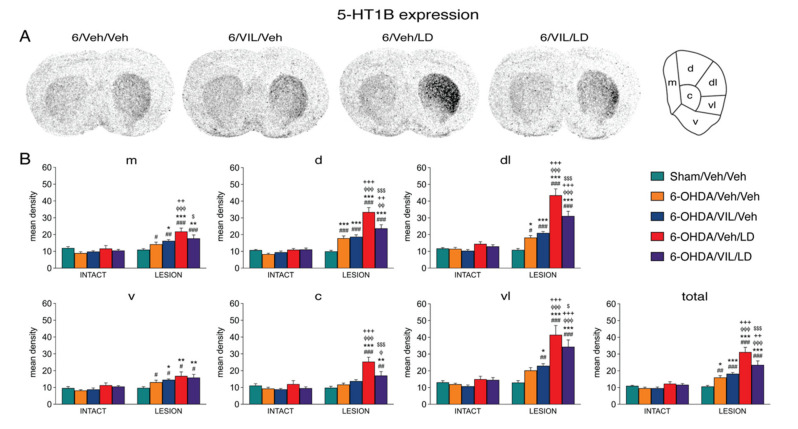
Vilazodone inhibited the L-DOPA-induced increase in 5-HT1B expression in the dopamine-depleted striatum. (**A**) Illustrations of film autoradiograms depict expression of 5-HT1B in sections from the mid-level striatum in rats with a 6-OHDA lesion (right hemisphere) followed by repeated vehicle (6-OHDA/Veh/Veh), vilazodone (10 mg/kg) + vehicle (6-OHDA/VIL/Veh), vehicle + L-DOPA (5 mg/kg) (6-OHDA/Veh/LD), or vilazodone + L-DOPA treatment (6-OHDA/VIL/LD). Animals were killed 60 min after the L-DOPA injection. (**B**) Mean density values (mean ± SEM) for 5-HT1B expression in the striatum on the intact side or the side of the lesion in the sham lesion controls (Sham/Veh/Veh) and the 6-OHDA/Veh/Veh, 6-OHDA/VIL/Veh, 6-OHDA/Veh/LD, and 6-OHDA/VIL/LD groups, measured in the six sectors and the total middle striatum (upper right). m, medial; d, dorsal; dl, dorsolateral; vl, ventrolateral; c, central; v, ventral. ^#^ *p* < 0.05, ^##^ *p* < 0.01, ^###^ *p* < 0.001 vs. same group on the intact side; * *p* < 0.05, ** *p* < 0.01, *** *p* < 0.001 vs. Sham/Veh/Veh; ^φ^ *p* < 0.05, ^φφ^ *p* < 0.01, ^φφφ^ *p* < 0.001 vs. 6-OHDA/Veh/Veh; ^+^ *p* < 0.05, ^++^ *p* < 0.01, ^+++^ *p* < 0.001 vs. 6-OHDA/VIL/Veh; ^$^ *p* < 0.05, ^$$$^ *p* < 0.001 vs. 6-OHDA/Veh/LD.

Figure 2 and Figure 3 depict the effects of the 6-OHDA lesion and drug treatments on the expression of dynorphin and enkephalin in the mid-level striatum. The gene regulation effects in the rostral and caudal striatum were similar (data are not shown). Consistent with our previous findings [33] and a long literature (e.g., [53]), animals with a loss of striatal TH immunoreactivity ipsilateral to the 6-OHDA lesion of 88% or greater showed decreased expression of dynorphin (Figure 2) and increased expression of enkephalin (Figure 3) in the striatum on the side of the lesion (6-OHDA/Veh/Veh). Dopamine depletion (i.e., loss of TH signal; Figure 1) and depletion-induced changes in gene expression were relatively uniform throughout the striatum (Figure 2, Figure 3 and Figure 4).

**Figure 5:**
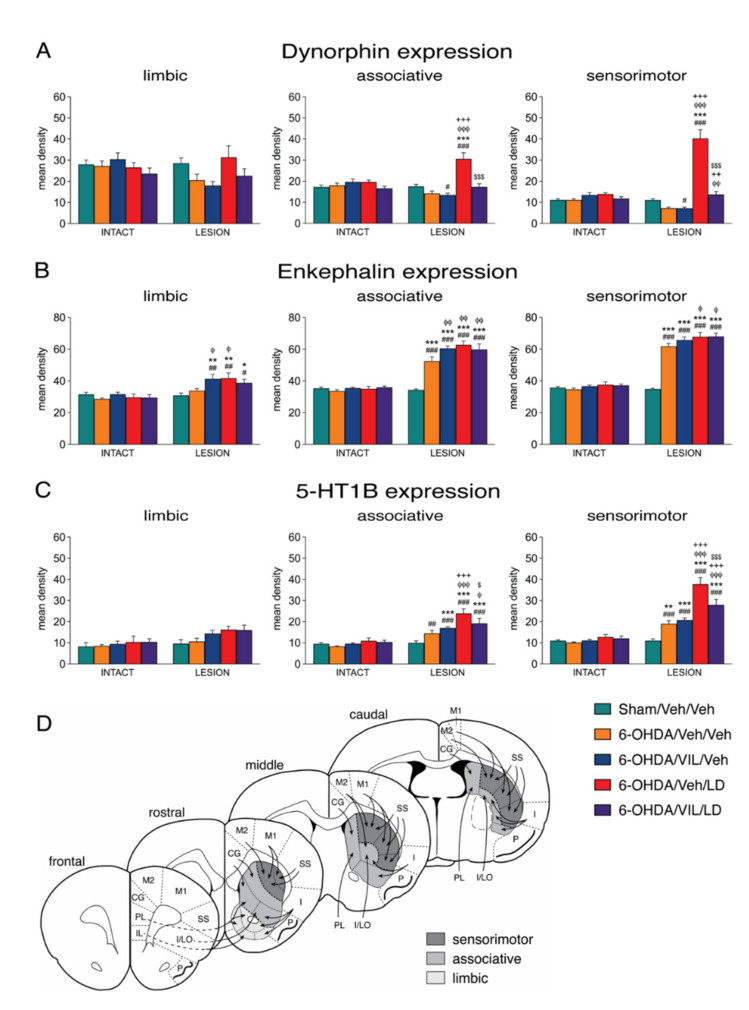
Summary of effects on striatal gene regulation in pooled limbic, associative, and sensorimotor sectors. Mean density values (mean ± SEM) for dynorphin (**A**), enkephalin (**B**), and 5-HT1B expression (**C**) in sectors from rostral, middle, and caudal levels pooled are shown for groups with a sham lesion (Sham/Veh/Veh) or a 6-OHDA lesion followed by repeated vehicle (6-OHDA/Veh/Veh), vilazodone + vehicle (6-OHDA/VIL/Veh), vehicle + L-DOPA (6-OHDA/Veh/LD), or vilazodone + L-DOPA treatment (6-OHDA/VIL/LD). Vilazodone inhibited the L-DOPA-induced increase in dynorphin and 5-HT1B expression in associative and sensorimotor sectors of the dopamine-depleted striatum, but had no effect on enkephalin expression in combination with L-DOPA. Vilazodone alone (6-OHDA/VIL/Veh) tended to further increase enkephalin expression over the lesion-induced increase (6-OHDA/Veh/Veh), mostly in associative sectors. (**D**) Schematic diagram of the striatal sectors used for measuring gene expression, categorized as limbic (nucleus accumbens), associative, and sensorimotor, with their simplified cortical inputs ([61,62]). ^#^ *p* < 0.05, ^##^ *p* < 0.01, ^###^ *p* < 0.001 vs. same group on the intact side; * *p* < 0.05, ** *p* < 0.01, *** *p* < 0.001 vs. Sham/Veh/Veh; ^φ^ *p* < 0.05, ^φφ^ *p* < 0.01, ^φφφ^ *p* < 0.001 vs. 6-OHDA/Veh/Veh; ^++^ *p* < 0.01, ^+++^ *p* < 0.001 vs. 6-OHDA/VIL/Veh; ^$^ *p* < 0.05, ^$$$^ *p* < 0.001 vs. 6-OHDA/Veh/LD.

Repeated L-DOPA treatment (6-OHDA/Veh/LD) produced a dramatic increase in dynorphin expression in the dopamine-depleted striatum on all three rostrocaudal levels (Figure 2 and Figure 5). Overall, after L-DOPA treatment, significantly increased dynorphin mRNA levels compared to the sham lesion group (6-OHDA/Veh/LD vs. Sham/Veh/Veh) were present in 16 of the 23 striatal sectors, and compared to the lesion-only group (6-OHDA/Veh/LD vs. 6-OHDA/Veh/Veh), in 18 of the 23 sectors (Figure 2 and Figure 5).

The increase in dynorphin expression displayed a distinct medial-lateral gradient (Figure 2A). The increase was minor in medial and ventral striatal sectors and was maximal in dorsal and lateral (“sensorimotor”) sectors on all three rostrocaudal levels (Figure 2 and Figure 5). This effect was also minimal or absent in sectors of the nucleus accumbens (pooled sectors, “limbic”; Figure 5). Therefore, the most pronounced L-DOPA-induced increase in dynorphin expression in the sensorimotor striatum markedly contrasted with the fairly uniform distribution of dopamine depletion (loss of TH signal) and dopamine depletion only-induced changes in dynorphin expression (Figure 1 and Figure 2), confirming our earlier findings [33]. 

The 6-OHDA lesion alone (6-OHDA/Veh/Veh vs. Sham/Veh/Veh) produced significantly increased enkephalin expression in 18 of the 23 striatal sectors on all three rostrocaudal levels, with lesser effects in ventral sectors (Figure 3 and Figure 5). Repeated L-DOPA treatment (6-OHDA/Veh/LD vs. 6-OHDA/Veh/Veh) caused a modest, but statistically significant, further increase in enkephalin expression in nine of these sectors, an effect that was most robust in pooled “associative” sectors (Figure 5). 

### 3.3. Effects of Vilazodone on Dynorphin and Enkephalin Expression after Dopamine Depletion and L-DOPA Treatment

Vilazodone strongly attenuated the L-DOPA-induced increase in dynorphin expression throughout the striatum. Thus, the vilazodone + L-DOPA-treated group displayed significantly lower levels of dynorphin mRNA compared to the L-DOPA only-treated group (6-OHDA/VIL/LD vs. 6-OHDA/Veh/LD) in 16 of the 23 sectors (Figure 2 and Figure 5). However, vilazodone did not completely eliminate the L-DOPA-induced increase in dynorphin expression, as the vilazodone + L-DOPA-treated group showed significantly higher levels of dynorphin expression than the 6-OHDA lesion-only group (6-OHDA/VIL/LD vs. 6-OHDA/Veh/Veh) in 4 of the 23 sectors (Figure 2 and Figure 5). This was observed in the dorsolateral sector on the rostral level (not shown) and the dorsal, dorsolateral, and ventrolateral sectors on the middle level (Figure 2), that is, in the sectors with the greatest L-DOPA-induced increase in dynorphin expression (sensorimotor sectors; Figure 5). Indeed, vilazodone normalized dynorphin expression in that dynorphin mRNA levels were similar (*p* > 0.05) to those in the sham lesion controls (6-OHDA/VIL/LD vs. Sham/Veh/Veh) in 22 of the 23 striatal sectors.

In contrast to the strong impact of vilazodone on L-DOPA-induced dynorphin expression (see above), vilazodone alone had no significant effect on dynorphin expression, either on the lesion side (6-OHDA/VIL/Veh vs. 6-OHDA/Veh/Veh) or on the intact side (Figure 2 and Figure 5).

In contrast to dynorphin expression, enkephalin expression was minimally or not affected by vilazodone, either on the lesion side or on the intact side (Figure 3 and Figure 5). For example, on the lesion side, enkephalin expression did not differ significantly (*p* > 0.05) between the vilazodone + L-DOPA-treated group and the L-DOPA only-treated group (6-OHDA/VIL/LD vs. 6-OHDA/Veh/LD) in any but one of the 23 sectors (Figure 3 and Figure 5). Also in contrast to dynorphin expression, there was a tendency for *increased* enkephalin expression after vilazodone-only treatment (6-OHDA/VIL/Veh vs. 6-OHDA/Veh/Veh; Figure 3) on the lesion side (borderline significant in six sectors and thus more robust after pooling sectors; Figure 5). Again, no effects on enkephalin expression were seen on the intact side (Figure 3 and Figure 5).

### 3.4. Effects of Vilazodone on 5-HT1B Receptor Expression after Dopamine Depletion and L-DOPA Treatment

Consistent with our previous findings [33], dopamine depletion and repeated L-DOPA treatment produced differential changes in 5-HT1B mRNA expression in the striatum ipsilateral to the lesion. The lesion effects were widespread, present on all three rostrocaudal levels (for effects on the middle level, Figure 4; rostral and caudal effects are not shown). After dopamine depletion alone (6-OHDA/Veh/Veh vs. Sham/Veh/Veh), there was a statistically significant increase in 5-HT1B expression in 10 of the 23 sectors, with lesser or no effects in ventral sectors, including the nucleus accumbens (Figure 4 and Figure 5).

Repeated L-DOPA treatment produced a pronounced further increase in 5-HT1B expression (Figure 4). This increase was seen in comparison to lesion-only animals (6-OHDA/Veh/LD vs. 6-OHDA/Veh/Veh) in 15 of the 23 sectors and to sham lesion controls (6-OHDA/Veh/LD vs. Sham/Veh/Veh) in 18 sectors, on all three rostrocaudal levels. Regionally, these effects were again strongest in the dorsal and lateral (sensorimotor) striatum and weakest in the medial and ventral striatum, including the nucleus accumbens (Figure 4 and Figure 5) and, thus, matching the distribution of the L-DOPA-induced increase in dynorphin expression (Figure 3 and Figure 5).

Also similar to dynorphin expression, vilazodone inhibited the L-DOPA-induced increase in 5-HT1B expression throughout the striatum (Figure 4 and Figure 5). The vilazodone + L-DOPA-treated group displayed significantly lower 5-HT1B mRNA levels compared to the L-DOPA only-treated group (6-OHDA/VIL/LD vs. 6-OHDA/Veh/LD) in 11 of the 23 sectors. Again, vilazodone did not completely suppress the L-DOPA-induced increase in 5-HT1B expression; the vilazodone + L-DOPA-treated group showed significantly higher levels of 5-HT1B expression than the 6-OHDA lesion-only group (6-OHDA/VIL/LD vs. 6-OHDA/Veh/Veh) in 10 of the 23 sectors (Figure 4 and Figure 5). This effect was seen in the sectors with the greatest L-DOPA-induced increase in 5-HT1B expression (sensorimotor sectors; Figure 4 and Figure 5).

Again similar to dynorphin and in contrast to the pronounced impact of vilazodone on L-DOPA-induced 5-HT1B expression (see above), vilazodone alone had no significant effect on 5-HT1B expression, neither on the lesion side (6-OHDA/VIL/Veh vs. 6-OHDA/Veh/Veh) (with the exception of one sector; not shown) nor on the intact side (Figure 4 and Figure 5).

### 3.5. Effects of Vilazodone on Zif268 Expression after Dopamine Depletion and L-DOPA Treatment

**Figure 6:**
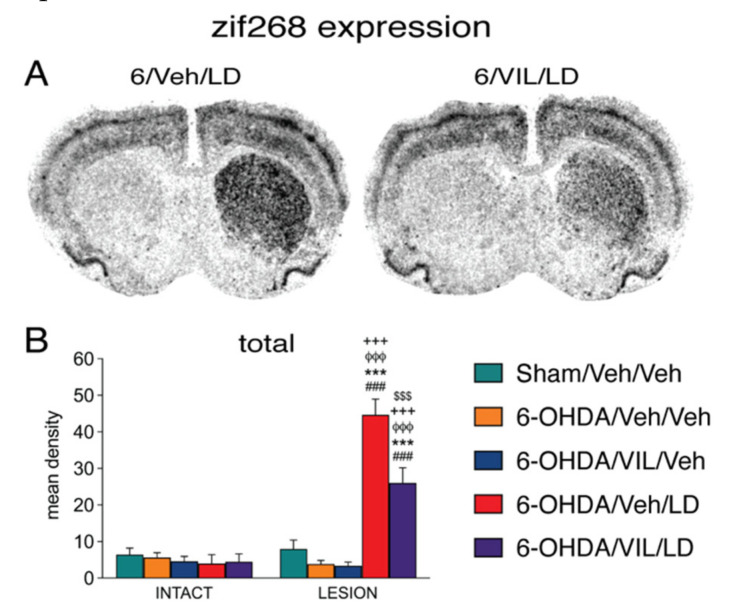
Vilazodone inhibited the L-DOPA-induced zif268 expression in the dopamine-depleted striatum. (**A**) Illustrations of film autoradiograms depict the expression of zif268 in sections from the mid-level striatum in rats with a 6-OHDA lesion (right hemisphere) followed by repeated vehicle + L-DOPA (6-OHDA/Veh/LD) or vilazodone + L-DOPA treatment (6-OHDA/VIL/LD). Rats were killed 60 min after the L-DOPA injection. (**B**) Mean density values (mean ± SEM) for zif268 expression in the striatum on the intact side or the side of the lesion in the sham lesion controls (Sham/Veh/Veh) and the 6-OHDA/Veh/Veh, 6-OHDA/VIL/Veh, 6-OHDA/Veh/LD, and 6-OHDA/VIL/LD groups, measured in the total middle striatum. ^###^ *p* < 0.001 vs. same group on the intact side; *** *p* < 0.001 vs. Sham/Veh/Veh; ^φφφ^ *p* < 0.001 vs. 6-OHDA/Veh/Veh; ^+++^ *p* < 0.001 vs. 6-OHDA/VIL/Veh; ^$$$^ *p* < 0.001 vs. 6-OHDA/Veh/LD.

The treatment effects on zif268 expression in the middle striatum are shown in Figure 6 (data from rostral and caudal levels were similar and are not shown). L-DOPA produced a pronounced increase in zif268 expression, an effect that was fairly uniform throughout the striatum (Figure 6). Vilazodone attenuated but did not eliminate the L-DOPA-induced increase in zif268 expression (Figure 6). Thus, the vilazodone effect on L-DOPA-induced zif268 expression mimicked that on dynorphin and 5-HT1B expression.

## 4. Discussion

L-DOPA-induced dyskinesia is associated with aberrant neuronal activity and altered gene regulation in striatal projection neurons produced by the L-DOPA treatment. These changes in gene regulation are thought to be driven by supraphysiological dopamine release from serotonin terminals. The present study used a rat model of L-DOPA-induced dyskinesia to investigate the effects of vilazodone, an SSRI/5-HT1A receptor partial agonist, on L-DOPA-induced changes in gene regulation in striatal projection neurons and behavior. Our main findings showed that a dose of vilazodone that suppressed the development of L-DOPA-induced dyskinesia also attenuated L-DOPA-induced gene regulation in direct pathway neurons (dMSNs), as demonstrated by the inhibition of the L-DOPA-induced increase in expression of the cell type marker dynorphin. Vilazodone also inhibited L-DOPA-induced expression of 5-HT1B and zif268, which also occurs in dMSNs. In contrast, no effects of vilazodone on the expression of enkephalin (a marker for iMSNs), nor on gene regulation in the intact striatum, were found. Overall, these results indicate that vilazodone co-administration selectively attenuated L-DOPA-induced gene regulation in dMSNs, and suggest that the antidyskinetic effects of vilazodone are mediated via preventing such L-DOPA-induced pathophysiological changes in the direct pathway neurons.

### 4.1. Vilazodone Attenuates L-DOPA-Induced Dyskinesia Without Inhibiting Its Prokinetic Effects

A number of previous studies have found that treatments with SSRIs and 5-HT1A agonists attenuate L-DOPA-induced dyskinesia in animal models (e.g., [18,36,37,38,39,40]). As these agents temper serotonin release, it is taken that at least part of this behavioral effect is due to attenuation of abnormal dopamine release from serotonin terminals [14,19,65]. However, some of these agents, especially at higher doses, also impair therapeutic (prokinetic) effects of L-DOPA or have other side effects [41,42,47], questioning the usefulness of this pharmacological approach. In contrast, a recent study showed that vilazodone, which combines SSRI and 5-HT1A partial agonist properties [49,50,51], given in conjunction with L-DOPA inhibited the development and expression of L-DOPA-induced dyskinesia while leaving L-DOPA’s prokinetic effects intact, as assessed by the forepaw stepping test [52].

Our present findings confirm and extend these previous results. We demonstrated that the dose of 10 mg/kg of vilazodone, which displayed a 100% occupancy of serotonin transporter sites in several brain regions [49], almost completely prevented the development of dyskinesia (measured as AIMs) during the course of a three-week, 13-injection L-DOPA treatment. Moreover, our results also showed that vilazodone suppressed all major subtypes of L-DOPA-induced dyskinesia in this rat model (axial, forelimb, orolingual AIMs), as well as L-DOPA-induced contraversive turning behavior. Importantly, also consistent with previous results [52], this vilazodone treatment did not impair the improved forepaw stepping, a simple motor response that is sensitive to dopamine loss and replacement by L-DOPA [55,66]. It has been shown that, while eliminating the dopamine spikes that produce L-DOPA-induced dyskinesia, this vilazodone + L-DOPA treatment elevates dopamine levels in the denervated striatum to some degree [52], thus presumably facilitating the execution of this simple behavior.

However, our findings also showed that more complex sensorimotor behavior (orienting to a novel environment [67]) was not normalized. The vilazodone + L-DOPA-treated animals still showed marked turning asymmetries in direction ipsiversive to the lesion in the open-field test, which reflects a dopamine denervation-induced sensorimotor neglect on the body surface contralateral to the dopamine lesion (e.g., [60,67,68]). Thus, while simple motor responses, such as stepping, were improved by this treatment, more complex sensorimotor integration might remain impaired.

### 4.2. Vilazodone Inhibits L-DOPA-Induced Increase in Dynorphin Expression (dMSNs)

Increased dynorphin expression after dopamine depletion followed by dopamine agonist treatments, including L-DOPA, is one of the best established molecular changes in striatal projection neurons (e.g., [31,32,69,70,71,72]). As dynorphin is selectively expressed by D1 receptor-containing dMSNs [53], this dramatic increase in gene expression reflects the aberrant molecular signaling involving supersensitive D1 receptors in dMSNs following dopamine depletion and L-DOPA treatment [25]. This altered gene regulation, likely involving thousands of genes (e.g., [28,29,30]), is thought to be critically important for the development of dyskinesia [9,25].

Consistent with our previous findings [33] and those of others (e.g., [31,32]), our results showed decreased expression of dynorphin after the 6-OHDA lesion alone and markedly increased expression after the subsequent repeated L-DOPA treatment. As described before [33], the L-DOPA-induced increase in dynorphin expression was widespread throughout the striatum but displayed distinct regional variations. The increase was most pronounced in the sensorimotor striatum and was lower or minimal in medial (associative) and ventral (limbic) sectors. Previous work showed that this distinct pattern of sensorimotor-dominant gene regulation after L-DOPA treatment is not specific for dynorphin, but extends to other genes as well, for example, the transcription factors dFosB and JunB [32,73], the serotonin receptor 5-HT1B ([33]; present study), and other genes [74,75]. These findings thus highlight the close association between abnormal signaling in dMSNs of the sensorimotor striatum and dyskinesia, in general [76,77,78,79].

Our findings demonstrate that vilazodone inhibited the L-DOPA-induced increase in striatal dynorphin expression. This result is in agreement with a recent study using RT-PCR to measure the effects of vilazodone on striatal dynorphin expression (in total striatal tissue [52]), as well as studies investigating other antidyskinetic agents [44,80]. Our present mapping study showed that this effect occurred throughout the striatum. It was most striking in the sensorimotor striatum but was also present in the associative striatum, which, although less affected by L-DOPA, nevertheless appears to contribute to L-DOPA-induced dyskinesia (for discussion [33]). The associative striatum has also been linked to non-motor side effects of L-DOPA treatment, such as impulsive/compulsive behaviors and visual hallucinations [81]. Attenuated gene regulation by vilazodone in both the sensorimotor and associative striatum may thus be essential to effectively reduce various L-DOPA-induced adverse effects.

Interestingly, after vilazodone + L-DOPA treatment, dynorphin mRNA levels were not entirely reduced to lesion-only levels, but were similar to normal levels (sham lesion controls) and, in this sense, were “normalized” (most clearly seen in the sensorimotor striatum). It is unclear whether a higher dose of vilazodone would have prevented increased dynorphin expression entirely. It is interesting to speculate that this normalization of gene regulation reflected the (partially) increased dopamine steady-state levels [52] and normalized corticostriatal synaptic activation (Altwal et al., in preparation) produced by this vilazodone dose, thus enabling some normalization of behavior in addition to preventing L-DOPA-induced dyskinesia.

### 4.3. Vilazodone Does Not Inhibit Enkephalin Expression (iMSNs)

Another well-established gene regulation effect of dopamine depletion is increased enkephalin expression in iMSNs (e.g., [69,71,82,83,84]; for review, [53]). L-DOPA tends to further increase enkephalin expression, but our direct comparison showed that L-DOPA had a more modest effect on enkephalin expression than on dynorphin expression (e.g., [33]; present results). In our previous study, we found a minor, but statistically significant, further increase in enkephalin expression in several striatal sectors, after repeated L-DOPA treatment [33]. Here, we obtained a similar but less robust effect, perhaps due to the shorter L-DOPA treatment in the present study (13 injections over 3 weeks) compared to the previous study (18 injections over 4 weeks [33]).

Our results showed that, in marked contrast to dynorphin, vilazodone did not affect enkephalin expression after dopamine depletion + L-DOPA treatment. Again in contrast to dynorphin expression, after dopamine depletion alone, vilazodone tended to increase enkephalin mRNA levels, at least in the limbic and associative striatum, which displayed a lesser increase in enkephalin expression by the dopamine depletion.

The reasons for these differential effects remain unclear. Gene regulation of enkephalin is more complex than that of dynorphin, with a significant impact by cholinergic activity [85] and an antagonistic regulation by cortical glutamate vs. striatal dopamine ([86]), which each may be under distinct serotonin receptor control. Besides, it has been shown that continuous (D2) dopamine receptor stimulation is required to reverse dopamine lesion-induced enhanced enkephalin expression (e.g., [69,83]), while intermittent (D1) dopamine receptor stimulation is sufficient to reverse changes in dynorphin expression (e.g., [69]).

Irrespective of the underlying mechanisms, our findings demonstrate that vilazodone had a markedly differential impact on gene regulation in dMSNs vs. iMSNs and that attenuation of the former was associated with the suppression of L-DOPA-induced dyskinesia. Thus, these results support the notion that vilazodone’s antidyskinetic properties are mediated by direct pathway-dependent mechanisms (reducing aberrant dMSN signaling), in the dopamine-depleted striatum. However, given that enkephalin expression (iMSN signaling) was not affected (not normalized), it is not surprising that vilazodone did not appear to completely normalize more complex behavior (sensorimotor integration).

### 4.4. Vilazodone Inhibits L-DOPA-Induced Increases in 5-HT1B and Zif268 Expression

The effects of vilazodone on 5-HT1B and zif268 expression support the above conclusion of a selective impact of this serotonergic agent on gene regulation in dMSNs. The 5-HT1B serotonin receptor subtype is expressed in both types of striatal projection neurons—dMSNs and iMSNs [29]. This receptor is dynamically regulated by a number of pharmacological treatments and experimental manipulations, for example, dopamine depletion and treatments with dopamine agonists, including psychostimulants and L-DOPA (cf. [33]). There is evidence that dopamine depletion and L-DOPA treatment differentially upregulate 5-HT1B in dMSNs vs. iMSNs [29]. Similar to enkephalin, dopamine depletion seems to preferentially increase 5-HT1B expression in iMSNs [29], with a rather uniform regional distribution, matching that of increased enkephalin expression ([33]; present results). On the other hand, similar to dynorphin, repeated L-DOPA treatment after dopamine depletion produces increased 5-HT1B expression selectively in dMSNs [29], with a regional distribution (sensorimotor dominant) that matches that of increased dynorphin expression ([33]; present results). Our previous findings showed that L-DOPA-induced increases in 5-HT1B expression correlated with the magnitude of L-DOPA-induced dyskinesia [33]. Underscoring this association between striatal gene regulation and behavior, our present results demonstrate that a dose of vilazodone that suppressed the development of L-DOPA-induced dyskinesia inhibited this L-DOPA-induced expression of 5-HT1B, presumably in dMSNs [29], while not inhibiting the lesion-induced increase in 5-HT1B, presumably in iMSNs.

Our findings on zif268 expression support a preferential effect of this serotonergic agent on dMSNs. Zif268 is a transcription factor (an immediate-early gene) that is induced by various dopamine agonists, including L-DOPA, and plays a role in several forms of neuroplasticity (cf. [86]). After dopamine depletion, dopamine receptor agonists dramatically induce zif268, selectively in dMSNs throughout the striatum [87], a reflection of supersensitive D1 receptor signaling. Our results demonstrate that vilazodone attenuated this abnormal zif268 response in the dopamine-depleted striatum. These findings thus also indicate a preferential impact of vilazodone on gene regulation in the D1 receptor-regulated direct pathway.

### 4.5. Possible Mechanisms Underlying the Effects of Vilazodone

The mechanisms by which vilazodone elicits its antidyskinetic action seem to involve a reduction of aberrant dMSN signaling following L-DOPA treatment. The attenuated increase in dynorphin, 5-HT1B, and zif268 expression is consistent with a vilazodone-induced inhibition of L-DOPA-derived dopamine release from striatal serotonin terminals.

Studies have shown that the serotonergic innervation of the striatum is essential for the development of L-DOPA-induced dyskinesia [13,16,18,88]. As monoaminergic cells, serotonin neurons contain the necessary cellular mechanisms to take up L-DOPA, convert it to dopamine, package it into vesicles, and then release it [15,89]. In advanced PD with a severe loss of striatal dopamine terminals, it is thought that serotonin neurons become the main source for dopamine release in the striatum. However, serotonin neurons lack the feedback control mechanism that is vital to regulate the release of dopamine (i.e., D2 autoreceptors), resulting in extreme fluctuations in extracellular dopamine levels following administration of L-DOPA (e.g., [12]).

Previous studies using SSRIs or 5-HT1A receptor full or partial agonists have demonstrated beneficial antidyskinetic effects of these drugs when combined with L-DOPA treatment (e.g., [18,37,38,40]). SSRIs increase extracellular serotonin levels, which activate 5-HT1A receptors and eventually reduce the activity of serotonin neurons in the DRN and dopamine release therefrom [40,90,91]. 5-HT1A receptor agonists (e.g., 8-OH-DPAT) and partial agonists (e.g., buspirone) produce a similar effect by directly activating 5-HT1A receptor in the DRN (e.g., [18,37,38]).

Similarly, vilazodone seems to convey its antidyskinetic effects, at least in part, via a 5-HT1A receptor-dependent mechanism, as blocking 5-HT1A receptors using a selective 5-HT1A receptor antagonist (WAY100635) inhibits the ability of vilazodone to reduce AIMs [52]. Together, these findings suggest that the antidyskinetic effects of vilazodone may be mediated through its direct and indirect activation of 5-HT1A autoreceptors, which would reduce the activity of serotonin neurons innervating the striatum and temper their dopamine release, thus reducing stimulation of supersensitive D1 receptors and concomitant abnormal molecular signaling.

However, vilazodone was administered systemically, which precludes conclusions regarding the sites of action; other mechanisms could thus also be involved. Serotonin receptors have a wide distribution throughout the brain [92] and modulate activity in various nodes of cortico-basal ganglia-thalamocortical circuits. For example, the cortex receives a prominent serotonin innervation, and cortical neurons express several 5-HT receptor subtypes (e.g., 5-HT1A, 5-HT2A, and others) (reviewed in [93]). Increased extracellular serotonin levels via the SSRI action of vilazodone may thus also modulate the excitability of cortical neurons by activating these 5-HT heteroreceptors. Given that aberrant facilitation of corticostriatal synaptic transmission is considered a major contributor to L-DOPA-induced dyskinesia (reviewed in [94]), moderating cortical neuronal activity, or glutamate release from corticostriatal terminals via 5-HT1A receptors located on these terminals [44], could be additional mechanisms underlying the vilazodone effects on dyskinesia.

Indeed, increased corticostriatal input (Altwal et al., in preparation) likely contributes to the increased striatal gene regulation associated with dyskinesia. We have hypothesized before [33] that the striking increase in dynorphin (and 5-HT1B) expression predominantly in the sensorimotor striatum (which markedly contrasts with the uniform loss of dopamine terminals/tyrosine hydroxylase throughout the striatum [33], present results) may at least in part reflect enhanced re-entrant corticostriatal input, as a consequence of abnormally enhanced D1 receptor-regulated striatal output in the direct pathway [95] and enhanced thalamocortical activity ([33], for discussion). Thus, 5-HT receptor-mediated dampening of cortical input may have contributed to vilazodone-induced inhibition of striatal gene regulation.

However, striatal gene regulation is under considerable influence of cortical glutamate also in the intact brain [86], yet vilazodone alone had no clear effect on striatal gene regulation, either in the dopamine-depleted or intact striatum. Therefore, a major contribution of dampened glutamate release per se to vilazodone-induced attenuation of dMSN gene regulation and dyskinesia is questionable, and interaction with abnormal dopamine release seems more likely. Further studies will have to investigate the specific mechanisms targeted by vilazodone.

## 5. Conclusions

Supraphysiological dopamine release from serotonin terminals in the striatum seems to drive L-DOPA-induced dyskinesia via abnormal gene regulation in striatal direct pathway neurons. In animal models, SSRIs and 5-HT1A receptor agonists independently have had beneficial antidyskinetic effects when given in conjunction with L-DOPA, presumably via tempering of such dopamine release; however, such agents can interfere with L-DOPA-induced prokinetic effects or may have other unwanted side effects (see above). Vilazodone, an FDA-approved antidepressant, has a unique pharmacological profile, combining SSRI and 5-HT1A receptor partial agonist properties. Our results in a rat model of PD show that vilazodone attenuates the development of L-DOPA-induced dyskinesia and selectively inhibits L-DOPA-induced aberrant gene regulation in striatal direct pathway neurons. These findings situate this multimodal serotonergic drug as a promising candidate for improving L-DOPA treatment and alleviate motor dysfunction in patients with PD.
